# Post–Myocardial Infarction Ventricular Septal Defect Percutaneous Closure

**DOI:** 10.1016/j.jaccas.2026.106864

**Published:** 2026-04-01

**Authors:** Shikha Jha, Parnian Soltani, Abdelkader Almanfi

**Affiliations:** a University of Wisconsin Madison, Madison, Wisconsin, USA; b Tehran Heart Center, Cardiovascular Disease Research Institute, Tehran University of Medical Sciences, Tehran, Iran; c Division of Cardiovascular Medicine, Baylor St Luke's Medical Center, Houston, Texas, USA

**Keywords:** access site, endovascular repair, myocardial infarction, percutaneous closure device, structural heart disease, treatment outcome, ventricular septal defect

## Abstract

**Background:**

Ventricular septal rupture is an uncommon but serious complication of late post–myocardial infarctions (MI) with an exceedingly high mortality. The American College of Cardiology and American Heart Association still advise immediate surgical closure of ventricular septal rupture.

**Case Summary:**

We described a novel minimally invasive transcatheter approach to post-MI ventricular septal defect (PMIVSD) closure through a venovenous loop for an 82-year-old woman.

**Discussion:**

A PMIVSD is typically treated surgically and offers reasonable outcomes in patients who survive an initial healing phase. Transcatheter approaches serve as a potential treatment option for select high-risk surgical candidates.

**Take-Home Messages:**

Transcatheter closure is a viable alternative in high-risk PMIVSD patients unsuitable for surgery. A venovenous loop offers a safe and effective route for percutaneous VSD closure.

## Visual Summary

**Figure:**
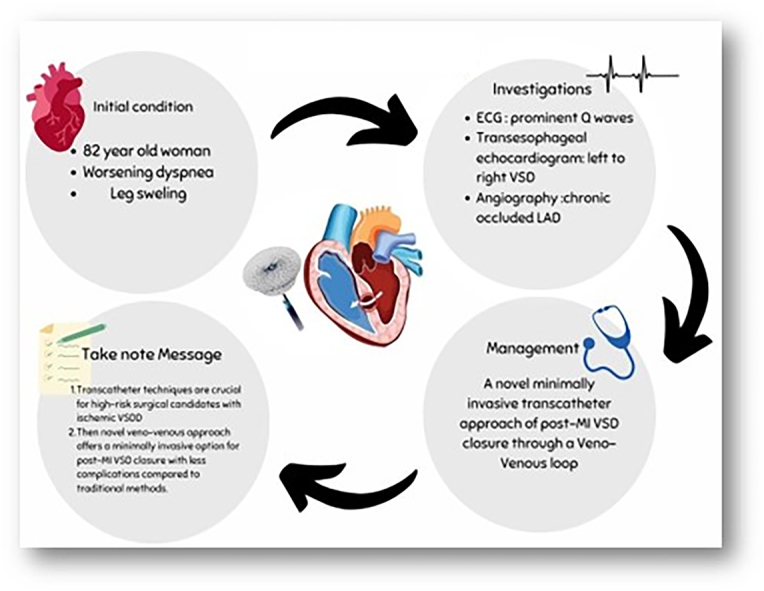

**Visual Summary:**
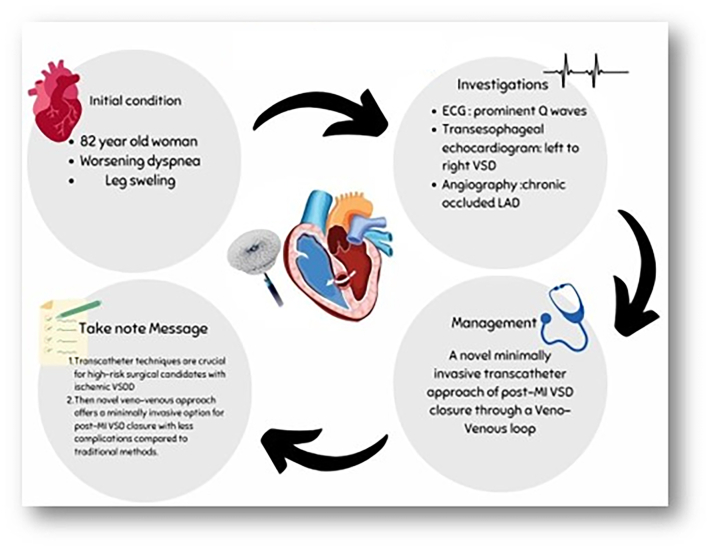
Xxx ECG = electrocardiogram; LAD = left anterior descending artery; MI = myocardial infarction; VSD = ventricular septal defect.

## History of Presentation

**Figure 1:**
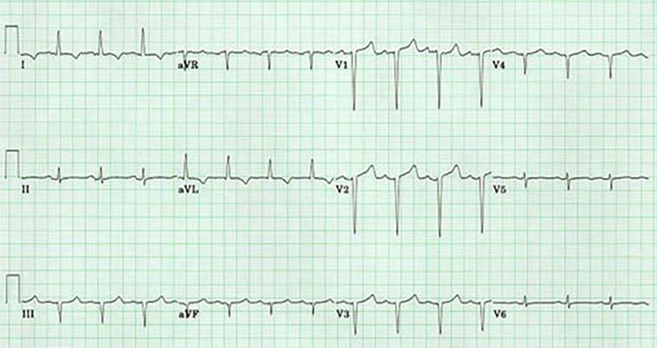
Electrocardiogram Initial electrocardiogram revealing sinus rhythm with prominent Q waves in leads II, aVF, and V_1_ to V_4_ accompanied by T-wave inversions in lead I and aVL.

An 82-year-old woman presented with symptoms of worsening dyspnea on exertion and leg swelling. Physical examination was significant for a systolic murmur. An electrocardiogram on admission revealed sinus rhythm with prominent Q waves in leads II, aVF, and V_1_ to V_4_ accompanied by T-wave inversions in leads I and aVL (Figure 1).

## Past Medical History

The patient had a known history of hypertension, hyperlipidemia, and diabetes. She reported experiencing an episode of chest pain during the early phase of the coronavirus disease 2019 pandemic. However, she preferred to remain at home to quarantine and limited her contact with the health care team during that time.

## Differential Diagnosis

The differential diagnosis includes systolic or diastolic heart failure, coronary artery disease, valvular disease, and myocarditis.

## Investigations

**Figure 2:**
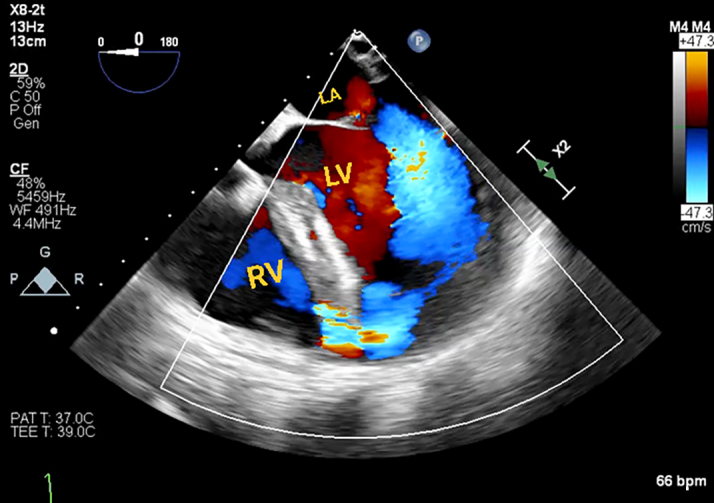
Transesophageal Echocardiogram Transesophageal echocardiogram with a newly diagnosed VSD. The green star is located superior to the area of the VSD. Color flow is depicted across the VSD. LA = left atrium; LV = left ventricle; RV = right ventricle; VSD = ventricular septal defect.

On the 2-dimensional echocardiogram, left ventricular (LV) function was measured at 55%, with mild concentric LV hypertrophy. The LV apex was noted to be aneurysmal and akinetic. The right ventricle was moderately dilated with severely reduced systolic function, and the pulmonary artery systolic pressure was 60 mm Hg. A new left-to-right ventricular septal defect (VSD) was discovered near the apical septum. It was further confirmed on the transesophageal echocardiogram (TEE) (Figure 2).

**Figure 3:**
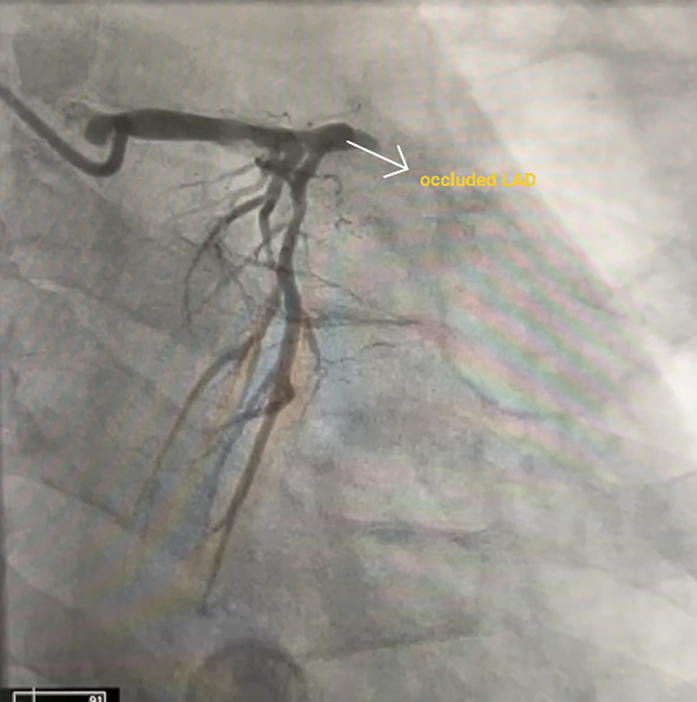
Coronary Angiogram Coronary angiogram revealing a proximally occluded left anterior descending artery (LAD) in the right anterior oblique caudal view.

Given her new-onset cardiomyopathy, she underwent right and left heart catheterization, which revealed a chronically occluded left anterior descending artery, consistent with chronic total occlusion, supporting the delayed diagnosis of prior nonrevascularized myocardial infarction (MI) (Figure 3).

The pulmonary/systemic flow ratio on right heart catheterization was notable for 1.7:1, with a pulmonary vascular resistance of 3.5 mm Hg·min/L.

## Management (Medical/Interventions)

The patient's symptoms of lower extremity edema and heart failure were likely due to right-sided heart failure secondary to the left-to-right shunt caused by the VSD. Her initial symptoms were managed with intravenous diuretic agents, an angiotensin-converting enzyme inhibitor, and a low dose of β-blockers.

Later, she was referred to our structural heart team for evaluation of percutaneous VSD closure as she was deemed a high-risk surgical candidate because of her frailty, location, and size of the defect.

Ischemic cardiomyopathy, specified by an aneurysmal akinetic apex, VSD, right ventricular failure, and pulmonary hypertension, was attributed to completed MI that went untreated because of limited health care contact during the coronavirus disease 2019 pandemic. The size of the VSD was 24 mm on the basis of TEE measurement. Given this background, we decided to use a dedicated post-MI VSD (PMIVSD) occluder.

An innovative approach was adopted using completely transvenous closure through a venovenous loop instead of the conventional arteriovenous loop because of the defect's location toward the apex.

**Figure 4:**
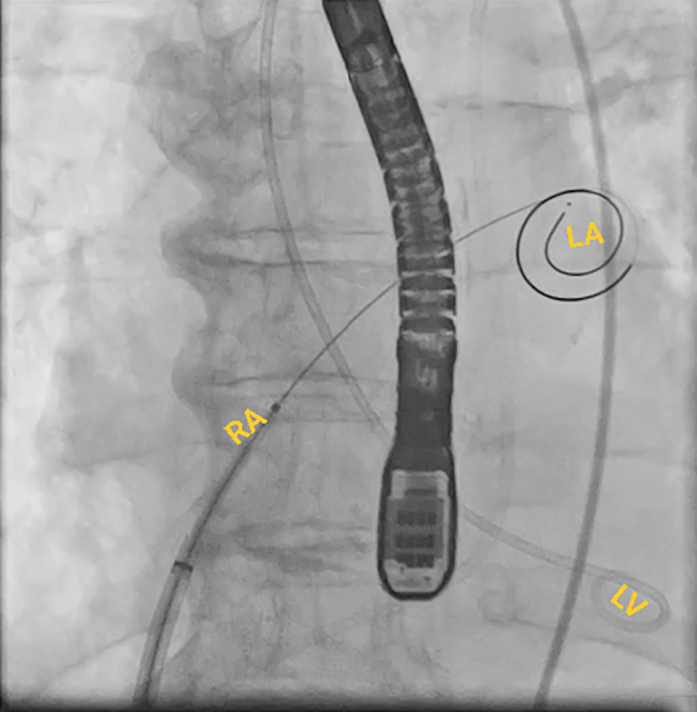
Fluoroscopic Image of the Procedure Fluoroscopic image of a Safari wire positioned in the LA through an atrial septal puncture. A transesophageal echocardiography probe is present in the center of the image. A pigtail is placed across the aortic valve. Abbreviations as in Figure 2.

**Figure 5:**
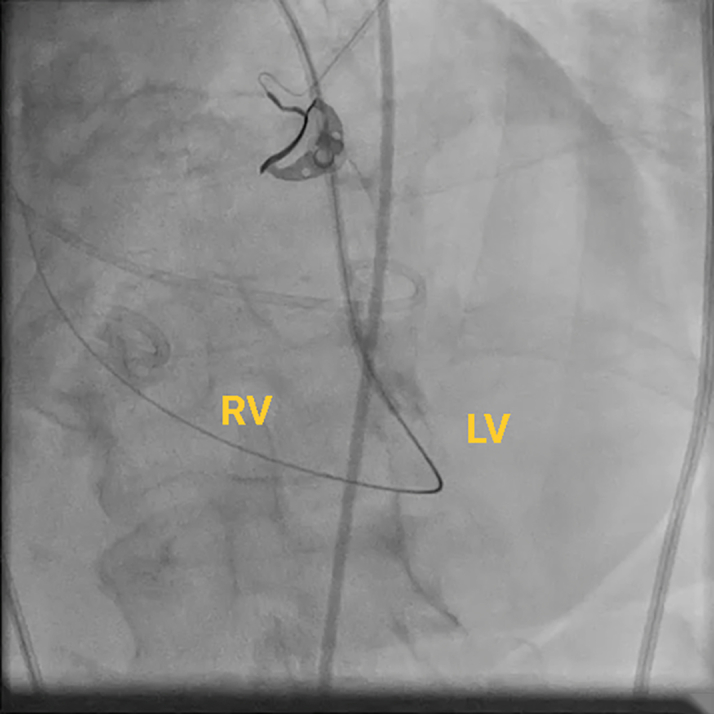
Fluoroscopic Image of the Procedure Fluoroscopic image of a guide catheter placed across the VSD from the LA via an atrial transseptal puncture. Abbreviations as in Figure 2.

**Figure 6:**
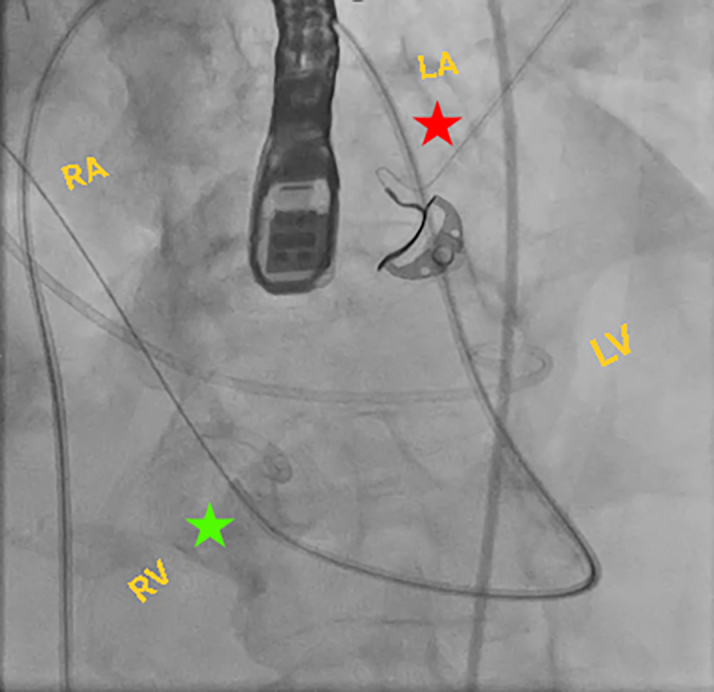
Fluoroscopic Image of the Procedure Fluoroscopic image of a guiding catheter crossing the mitral valve to the LV and advancing through the VSD (red star), with the glide wire extending into the outflow tract to be snared (green star). RA = right atrium; other abbreviations as in Figure 2.

**Figure 7:**
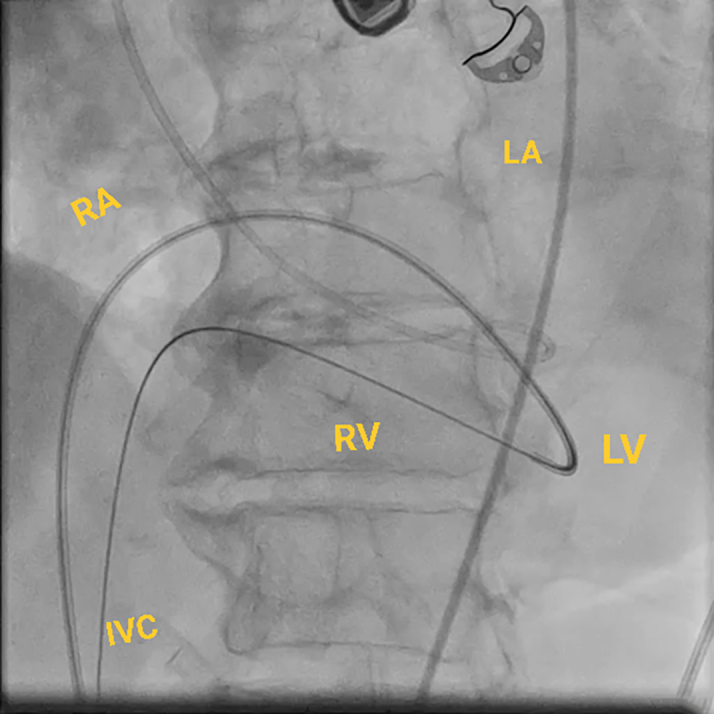
Fluoroscopic Image of the Procedure Fluoroscopic image of the snared wire. The wire course is as follows: from the RA to the LA via a transseptal puncture into the LV across the VSD into the RV and through the RA into the IVC, creating a venovenous loop. IVC = inferior vena cava; other abbreviations as in Figures 2 and 6.

**Figure 8:**
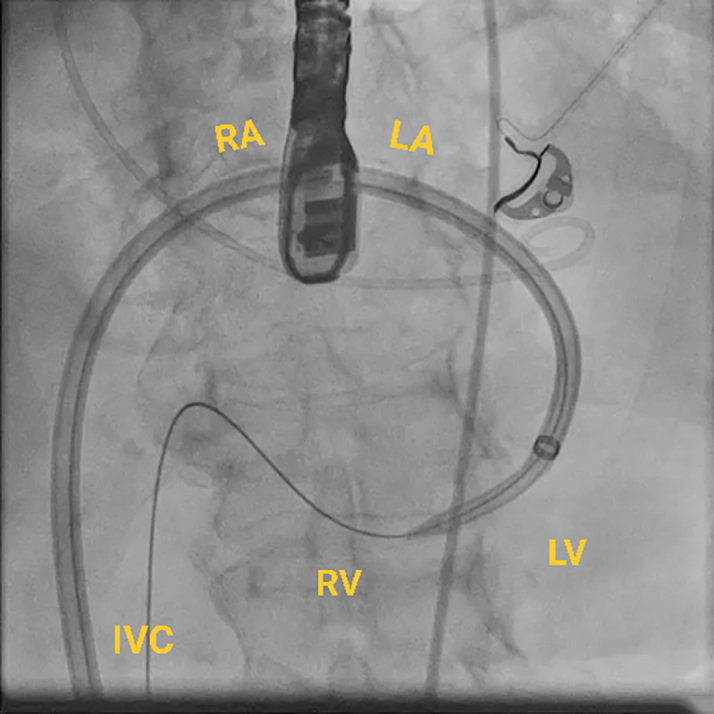
Fluoroscopic Image of the Procedure Fluoroscopic image of the delivery system for the post–myocardial infarction VSD occluder positioned across the VSD through a venovenous loop. Abbreviations as in Figures 2, 6, and 7.

**Figure 9:**
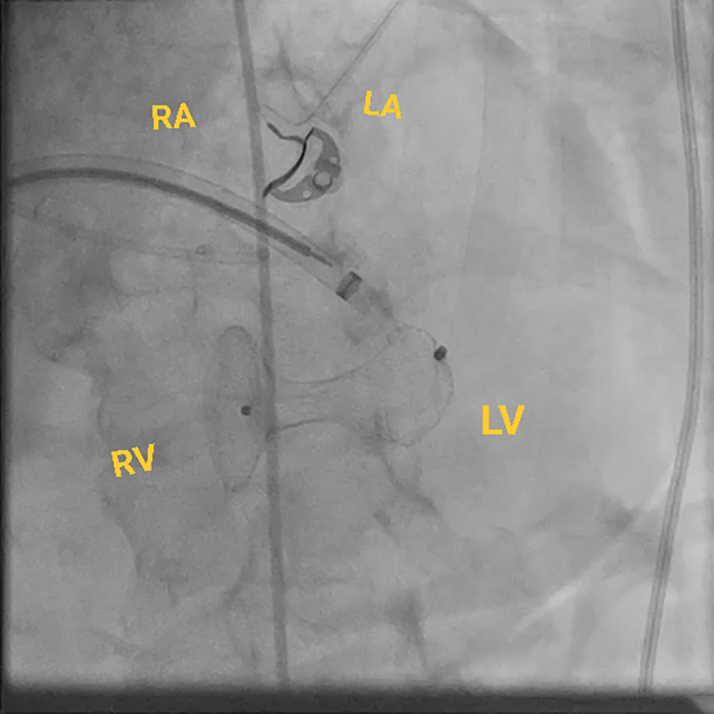
Fluoroscopic Image of the Procedure Fluoroscopic image of the final position of the post–myocardial infarction VSD occluder deployed across the VSD via a venovenous loop approach. Abbreviations as in Figures 2 and 6.

**Figure 10:**
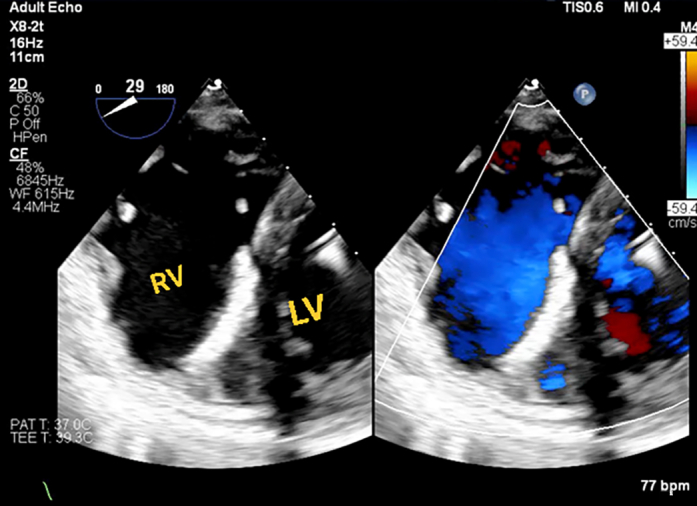
Final Transesophageal Echocardiogram Transesophageal echocardiogram at the end of the procedure showing no significant shunt. Abbreviations as in Figure 2.

This approach requires an atrial transseptal puncture to form the loop through the mitral valve rather than the traditional route through the aortic valve (Figure 4). The transseptal puncture was performed from the right common femoral vein using the Baylis system under fluoroscopic and TEE guidance, and a Baylis wire was positioned in the left atrium through an atrial septal puncture. The JR-4 guide catheter was used to direct the wire across the mitral valve into the LV. Then, we exchanged the Baylis wire with a long glide wire. The VSD was crossed with a JR-4 catheter and glide wire in the right ventricular outflow tract, and then the wire was snared using an ensnare introduced from the contralateral femoral vein. Finally, the wire was externalized from the venovenous loop. The post-MI occluder was successfully delivered and positioned within the defect via the atrial transseptal approach by forming a venovenous loop through the right and left femoral vein access. The procedure was guided by both fluoroscopy and TEE (Figure 5, Figure 6, Figure 7, Figure 8, Figure 9, Figure 10).

## Discussion

Ischemic ventricular septal rupture is an uncommon but devastating mechanical complication of late-presenting post-MIs, with an exceedingly high 30-day mortality rate of 94% when managed with medical therapy alone.^1^

A PMIVSD typically occurs as a bimodal pattern, with a higher incidence within the first 24 hours and then again 3 to 5 days after acute MI.^2^ The American College of Cardiology and American Heart Association still emphasize immediate surgical closure of ventricular septal rupture.^3^ Current indications for closure include left-to-right shunts with pulmonary/systemic flow ratio ≥1.5:1, pulmonary artery systolic pressure <50% of systemic pressure, pulmonary vascular resistance less than one-third of systemic resistance, or progressive aortic regurgitation.^4^

Therapeutic options remain challenging despite advancements in technology, primarily because of the necrotic borders of the defect.

Although surgical repair offers reasonable outcomes in patients who survive the initial healing phase of the PMIVSD, transcatheter approaches present a potential treatment option for select high-risk surgical candidates.^5^

In our case, we performed a minimally invasive approach with endovascular repair using a postinfarct muscular VSD occluder.

The Amplatzer PMIVSD device shows superior results and implantation success than do atrial or muscular VSD occluders.^6^

Once the wire was externalized, we deployed the right ventricular disc first, followed by the left ventricular disc. Some experts prefer the nontransseptal approach to ensure the LV disc expands first, optimizing septal opposition in left-to-right shunting.

Given the larger waist on the PIMVSD device—at least 4 mm wider than the diameter of the patient's VSD confirmed on TEE—we determined this device to be the most suitable according to cases reported in the U.S. registry data because necrotic tissue may cause further enlargement of the defect during implantation. Using preplanned gated computed tomography or 3-dimensional modeling is helpful for assessing the anatomy of the VSD, determining the optimal device size, and planning the procedural approach. However, we did not have computed tomography for this case and relied on TEE both preoperatively and during the procedure.

Although we used femoral access, many operators prefer using internal jugular access instead to achieve better coaxiality.

The presence of cardiogenic shock and VSD closure performed within 14 days are associated with a high mortality rate. In contrast, patients managed in the post–acute phase (>3 weeks) demonstrate a better survival rate.^7^

By using an approach through a venovenous loop, we reduced potential complications compared with the traditional aortic valve approach. Minimally invasive techniques, including percutaneous closure of VSDs using the transseptal approach, have been shown to provide safe and effective alternatives to traditional surgery, particularly in high-risk patients.^8^^,^^9^

## Follow-Up

**Figure 11:**
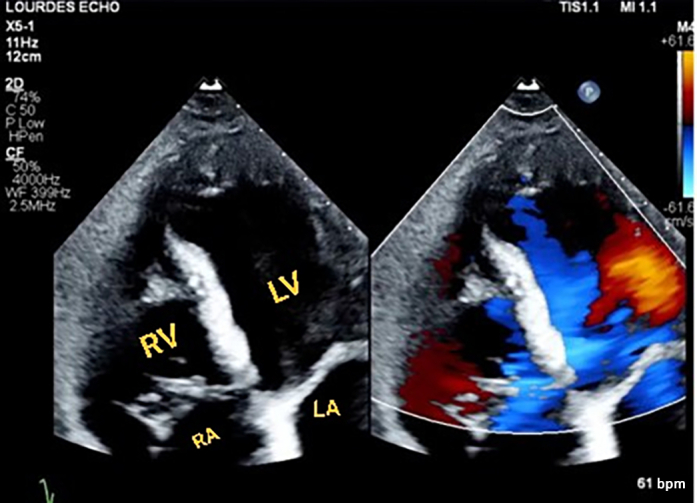
Follow-Up Transesophageal Echocardiogram A follow-up transesophageal echocardiogram recorded 1 month after the procedure revealed no residual shunt. Abbreviations as in Figures 2 and 6.

A follow-up TTE recorded 1 month after the procedure revealed no residual shunt (Figure 11). There were no postprocedural complications, and the patient was discharged home with resolution of her symptoms.

## Conclusions

We described a minimally invasive approach to the endovascular repair of a PMIVSD using a postinfarct muscular VSD occluder. The patient had late-presentation MI, which was initially undiagnosed, resulting in an ischemic VSD because of nonrevascularization. Transcatheter approaches are available as a treatment option for patients who are high-risk surgical candidates. The venovenous approach offers several advantages over the traditional method, including the elimination of arterial access, making it safer for patients with mechanical aortic valves, and providing better coaxiality and angulation for precise catheter and device delivery.

## Funding Support and Author Disclosures

Dr Almanfi has served as a national proctor, consultant, and speaker for Medtronic TAVR; a speaker and consultant for Abbott and Teleflex; and a speaker for Shockwave. All other authors have reported that they have no relationships relevant to the contents of this paper to disclose.

**Take-Home Messages**

• A venovenous loop approach offers a minimally invasive option for post– myocardial infarction VSD closure, reducing complications compared with traditional methods.
• Transcatheter techniques are crucial for high-risk surgical candidates with ischemic VSDs, expanding treatment options in structural heart disease.
